# Evaluation of the efficacy and surgical-related safety of neoadjuvant immunochemotherapy in advanced resectable none small cell lung cancer (NSCLC)

**DOI:** 10.3389/fonc.2023.1239451

**Published:** 2023-12-22

**Authors:** Qin Wang, Chen Qi, Jing Luo, Nan Xu, Mao-tian Xu, Yong Qiang, Chi Zhang, Yi Shen

**Affiliations:** ^1^ Department of Cardiothoracic Surgery, Jinling Hospital, Medical School of Nanjing University, Nanjing, China; ^2^ Department of Ultrasound, Jinling Hospital, Medical School of Nanjing University, Nanjing, China; ^3^ Department of Cardiothoracic Surgery, Jinling Hospital, School of Clinical Medicine, Nanjing Medical University, Nanjing, China; ^4^ Department of Cardiothoracic Surgery, Jinling Hospital, School of Medicine, Southeast University, Nanjing, China

**Keywords:** non-small cell lung cancer, neoadjuvant therapy, neoadjuvant immunotherapy, neoadjuvant immunochemotherapy, surgical-related safety

## Abstract

**Background:**

The emergence of immune checkpoint inhibitors (ICIs) has brought about a paradigm shift in the treatment landscape of non-small cell lung cancer (NSCLC). Despite the promising long-term survival outcomes and optimization of pathological complete response (cPR) demonstrated by various studies such as Impower010 and Checkmate-816, the effectiveness of neoadjuvant immunotherapy in advanced resectable NSCLC remains a subject of debate. Although previous research has explored the connection between the efficacy of neoadjuvant therapy and surgical-related safety, limited studies have specifically investigated the surgical-related safety of neoadjuvant immunotherapy. Therefore, our study aims to assess the efficacy and surgical-related safety of neoadjuvant immunotherapy in advanced resectable non-small cell lung cancer.

**Method:**

We conducted a retrospective study on a cohort of 93 patients with stage IIIA-IIIC NSCLC who underwent neoadjuvant therapy and surgical resection. Among them, 53 patients received neoadjuvant immunotherapy, 18 patients underwent neoadjuvant chemotherapy while the remaining 22 underwent neoadjuvant targeted therapy. The patients were separated into further groups according to their pathological type. Data analyses were performed using Mann-Whitney U test, chi-square test.

**Results:**

All patients were categorized into six distinct groups. Notably, the neoadjuvant immunotherapy squamous carcinoma group exhibited a favorable edge over the neoadjuvant targeted squamous carcinoma group concerning the duration of drainage tube indwelling and the extent of lymph node dissection. Furthermore, the neoadjuvant immunotherapy adenocarcinoma group outperformed neoadjuvant targeted therapy adenocarcinoma counterpart in terms of achieving complete pathological response (cPR). Simultaneously, the neoadjuvant immunotherapy adenocarcinoma group surpassed the neoadjuvant chemotherapy adenocarcinoma group in the incidence of hydrothorax. Nevertheless, no statistically significant disparities were noted between the neoadjuvant immunotherapy squamous carcinoma group and the neoadjuvant chemotherapy carcinoma group.

**Conclusion:**

Regarding surgical outcomes, neoadjuvant immunotherapy conferred notable advantages compared to conventional neoadjuvant chemotherapy and neoadjuvant targeted therapy for patients diagnosed with adenocarcinoma. In the case of squamous carcinoma, neoadjuvant immunotherapy exhibited superiority over neoadjuvant targeted therapy, although additional evidence is required to conclusively establish its precedence over neoadjuvant chemotherapy.

## Introduction

Lung cancer (LC) poses a significant global health challenge, with non-small cell lung cancer (NSCLC) accounting for approximately 80% to 85% of newly diagnosed cases. According to the cancer statistics worldwide, LC accounts for up to 2 million diagnosed cases and contributes to more than 1.5 million deaths annually. Despite of the persistent decline in the LC incidence and mortality among American and European countries, the number of newly diagnosed LC in China is still on the rise and the LC-related deaths rank first in the cancer-specific mortality (1–5). Surgical resection with curative intent continuous served as the principal treatment for resectable NSCLC and offed the best chance of cure (6). However, its five-year survival rate ranging from 36% to 60% for stage IIIA disease to stage IIA disease respectively remained dissatisfactory (7). Randomized trials had elucidated the latent advantage of adjuvant therapy versus surgery alone, albeit with only a modest 5% benefit was identified in the five-year overall survival (OS) (6, 8). Taken together, an urgent need for the advent of novel treatment was put forward (9).

Neoadjuvant therapy has emerged as a groundbreaking approach in the management of resectable NSCLC, offering significant advantages such as improved long-term survival and an increased likelihood of cPR (7). Theoretically, neoadjuvant therapy has the potential to enhance the resection rate and enable the timely elimination of subclinical micro-metastatic disease. Additionally, compared to traditional adjuvant therapy, neoadjuvant therapy demonstrates superior compliance due to the pathetic surgery recovery (10). A systematic review and meta-analysis conducted by the NSCLC Meta-analysis Collaborative Group delineated a potential 5% benefit in the 5-year survival rate when neoadjuvant chemotherapy was compared to surgery alone (11). In contrast, another systematic review showed no significant difference in the 5-year survival between the adjuvant and neoadjuvant therapy (12). Collectively, the advancement of neoadjuvant therapy holds promise in converting unresectable NSCLC into resectable cases through tumor shrinkage and early intervention in patients at high risk of developing tumor metastasis. Therefore, the safety of the surgical procedure is closely linked to the efficacy of neoadjuvant therapy, as improved tumor shrinkage results in a safer surgical approach and a reduced incidence of postoperative complications.

Despite the treatment landscape of NSCLC such as screening, minimally invasive techniques and radiotherapy had evolved mildly, the development of neoadjuvant therapy remained nascent during the past decades (13, 14). Immunotherapy with the detection of immune checkpoint inhibitors (ICIs) such as programmed cell death protein 1(PD-1), programmed cell death receptor-legend 1(PD-L1) and cytotoxic T-lymphocyte-associated protein 4(CTLA-4) stirred up ripples in the in the field of neoadjuvant therapy for NSCLC (15–17). Mechanistically, ICIs function by facilitating the recognition of tumor cells by host T-cells, leading to the activation of T-cells, the release of cytokines, and subsequent tumor cell elimination following blockade of inhibitory interactions by ICI antibodies. Tumors with larger sizes, which carry a higher antigen burden, are more likely to elicit a robust anti-tumor T-cell response and therefore stand to benefit more from immunotherapy (18–20). Various studies such as Impower010, Checkmate-816 and NEOSTAR had identified the significant advantage of neoadjuvant immunotherapy to date (21–23).

However, the current clinical landscape of first-line neoadjuvant immunotherapy for NSCLC is characterized by rapid variations both domestically and internationally, with no established guidelines for the selection of ICIs at present (24, 25). The ICIs approved by the U.S. Food and Drug Administration include pembrolizumab, atezolizumab, nivolumab, ipilimumab, and durvalumab. However, in China, there is a rapid differentiation in the ICIs utilized as first-line treatment for NSCLC, such as tislelizumab, sintilimab, and toripalimab (26–29). Moreover, previous studies on the efficacy of neoadjuvant immunotherapy have produced inconsistent results, which can be attributed to the limited sample sizes and the inclusion of NSCLC patients at different clinical stages, further intensifying the controversy surrounding these outcomes (30–33). Given the inconsistent findings from prior research and the absence of standardized guidelines for neoadjuvant treatment in advanced NSCLC, it becomes imperative to conduct additional studies to evaluate the efficacy of neoadjuvant immunotherapy in patients with advanced clinical stage NSCLC. Moreover, despite the strong association between the efficacy of neoadjuvant therapy and surgical-related safety, few studies have focused on the surgical-related safety following neoadjuvant immunotherapy. Therefore, our study aims to assess the efficacy and surgical-related safety of neoadjuvant immunotherapy in advanced NSCLC by comparing the outcomes of neoadjuvant therapy with immunotherapy to that of neoadjuvant therapy without immunotherapy in patients diagnosed with clinical stages IIIA-IVA NSCLC.

## Method

### Patient characteristics

A cohort of 104 NSCLC patients (≥18 years) diagnosed with a clinical stage ranging from IIIA to IVA were included. These patients underwent neoadjuvant therapy followed by surgical resection at the Department of Oncology and Cardiothoracic Surgery of Jinling Hospital between January 2016 and April 2023. Two patients were excluded from the study due to the use of neoadjuvant radiotherapy, which slightly increased the surgical risk due to the potential development of severe pleural adhesion (34). Nine patients were excluded with the clinical stage of IVA. This study was conducted in accordance with the Helsinki Declaration of 1964 and the latest version and met the ethical standards of the responsible committee on human experimentation of Jinling Hospital. Since this study was retrospective in nature, obtaining informed consent was not deemed necessary.

### Data collection

Patients’ data including age, gender, smoking history, clinical stage (cTNM), histological type of NSCLC and comorbid disease (cardiovascular disease, hypertension, diabetes mellitus) were extracted and analyzed. The surgery-related data included intraoperative blood loss, time of operation, indwelling time of drainage tube, total drainage volume, postoperative hospital stay, R0 resection, complete pathological response(cPR), the number of lymph node dissection fields, infection, sever pain, pneumothorax and hydrothorax. In consideration of the cPR, we collected the postoperative pathological report of all patients participated and cPR was referred to as no specific tumor remains among the widely drawing materials (35). The cTNM were according to the 8th American Joint Commission on Cancer (AJCC) Cancer Staging Manual (36). No immune-related adverse events(irAEs) of grade 3 or 4 was recorded in our study (37).

### Treatment strategy

A total of 93 patients was divided into 6 groups according to their treatment strategy and pathological type. 53 patients received 2-4 cycles of neoadjuvant immunotherapy combined with neoadjuvant chemotherapy, 38 of them came up with the pathological type of squamous carcinoma while the rest 15 were adenocarcinoma. 22 patients achieved 2-4 cycles of neoadjuvant targeted therapy, among them 6 were squamous carcinoma and 16 were adenocarcinoma. Among the 22 patients, 8 applied Gefitinib, 5 used Afatinib, 4 employed Almonertinib, 4 applied Osimertinib, 1 used Crizotinib. 18 received 2-4 cycles of neoadjuvant chemotherapy with 10 squamous carcinomas and 8 adenocarcinomas. All of the patients received an interval no less than four weeks between the neoadjuvant therapy and surgery (38). Due to the lack of guidelines regarding the selection of neoadjuvant immunotherapy, the diverse treatment approaches made it impractical to subgroup patients within the neoadjuvant immunochemotherapy group based on their distinct strategies. The neoadjuvant chemotherapy or neoadjuvant targeted therapy followed the National Comprehensive Cancer Network (NCCN)version 2.2023 guidelines NSCLC (39). Thoracoscopic minimally invasive surgery was performed on all 93 patients in a standardized manner, with lymph node dissection carried out according to established protocols. The postoperative pathological reports were reviewed and verified by pathologists with over 10 years of experience.

### Statistical analyses

Statistical analysis was performed using SPSS 26.0. The comparison of continuous variables was conducted using the Mann-Whitney U test, and the results were presented as the median (M) and interquartile range (IQR, P25-P50). For categorical variables, the chi-square test was utilized, and the findings were reported as the number (n) with the corresponding percentage (%). A p-value < 0.05 was considered statistically significant.

## Results

### Neoadjuvant immunotherapy squamous carcinoma group(n=38) neoadjuvant targeted therapy squamous carcinoma group (n=6)

**Figure 1:**
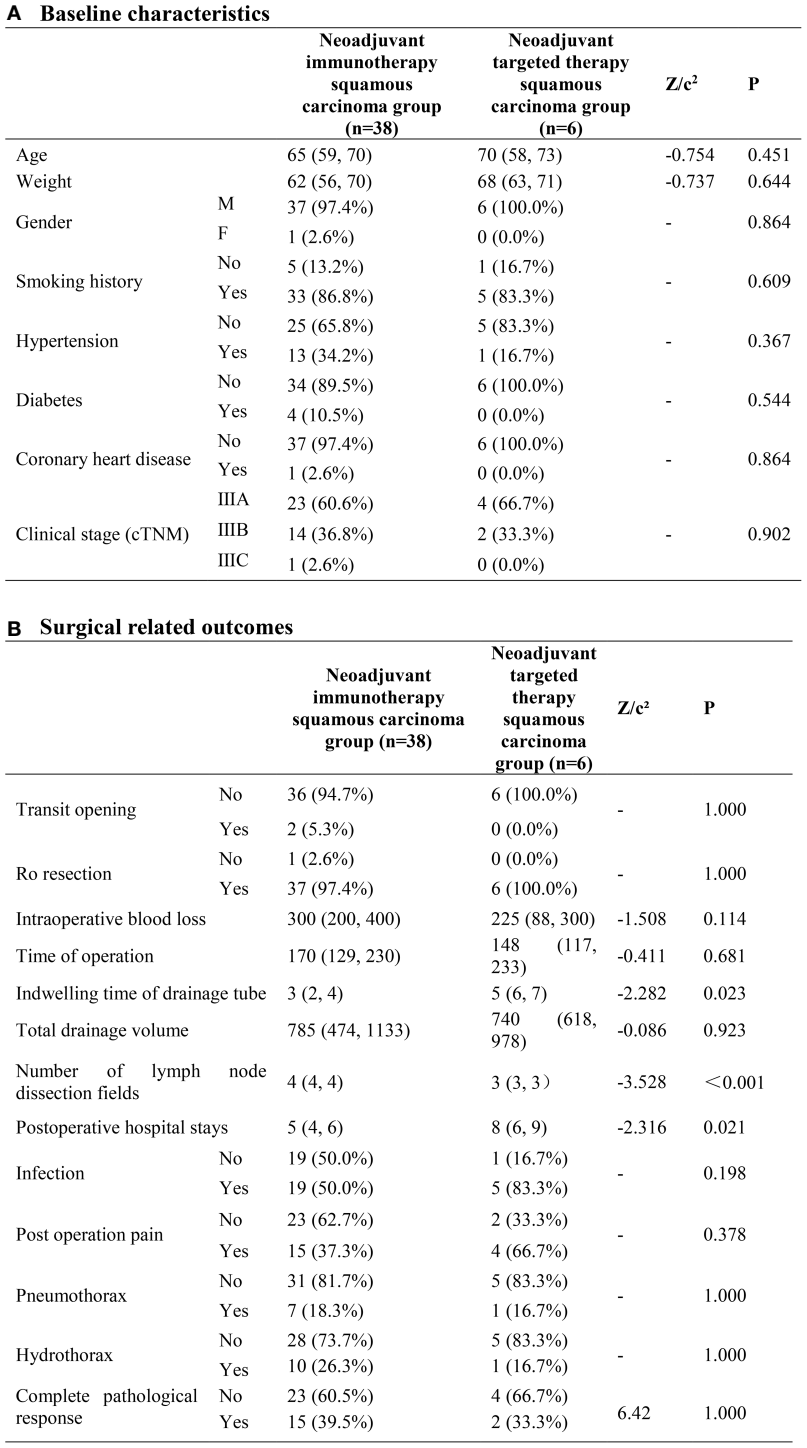
Neoadjuvant immunotherapy squamous carcinoma group and neoadjuvant targeted therapy squamous carcinoma group. **(A)** Baseline characteristics; **(B)** Surgical related outcomes.

In terms of demographics and baseline characteristics, no significant difference was observed within two groups. Through univariate analyses, neoadjuvant immunotherapy squamous carcinoma group exhibited advantages in terms of the indwelling time of drainage tube(P=0.023) and the number of lymph node dissection fields (P<0.001) (**Figure 1**).

### Neoadjuvant immunotherapy squamous carcinoma group (n=38) neoadjuvant chemotherapy squamous carcinoma group (n=10)

**Figure 2:**
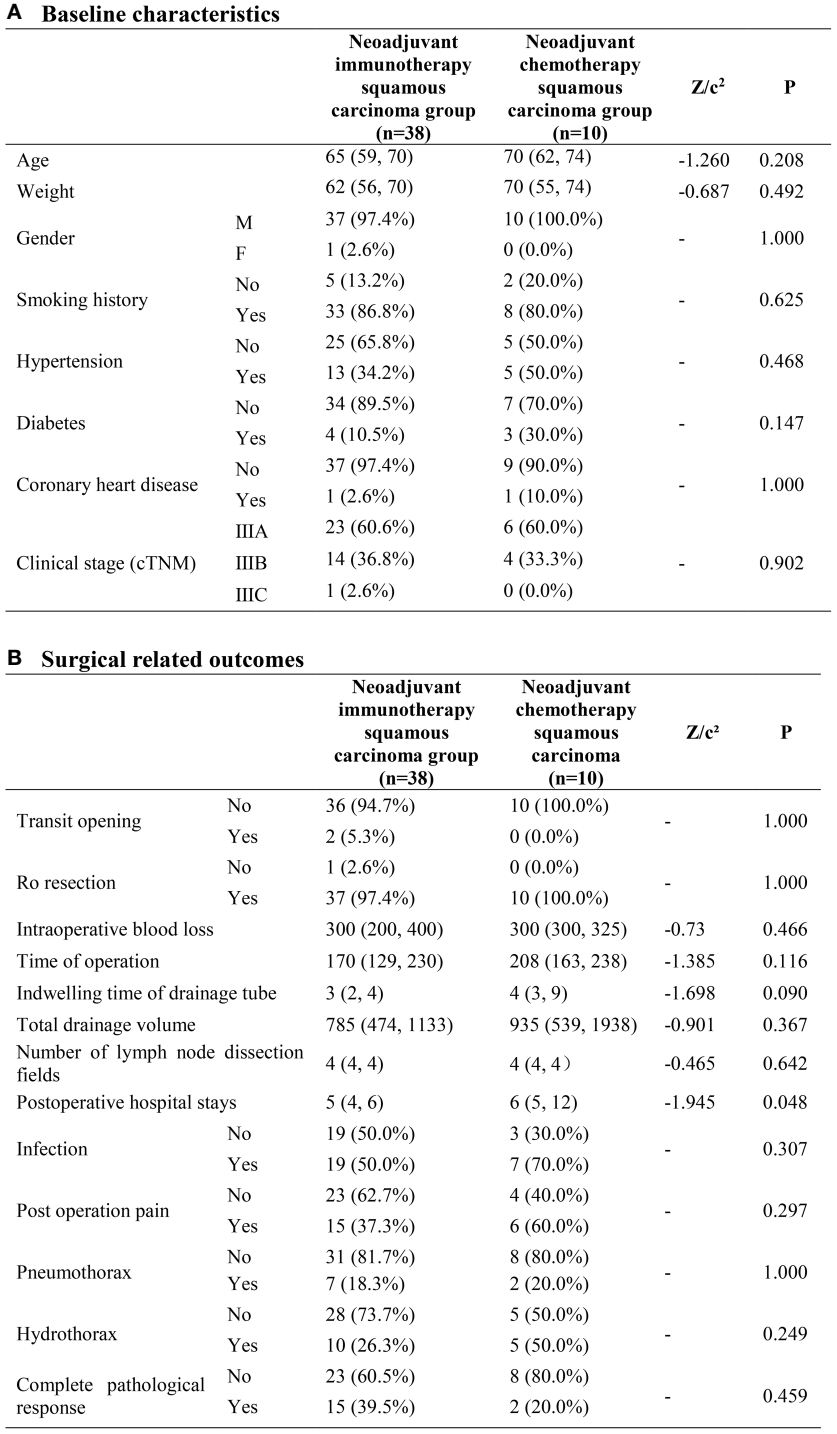
Neoadjuvant immunotherapy squamous carcinoma group and neoadjuvant chemotherapy squamous carcinoma group. **(A)** Baseline characteristics; **(B)** Surgical related outcomes.

No significant difference was found between the two groups with regards to both baseline characteristics and surgical related outcomes. The cPR rate was 39.5% in the neoadjuvant immunotherapy squamous carcinoma group and 20.0% in the neoadjuvant chemotherapy group, however without heterogeneity (P=0.459) (**Figure 2**).

### Neoadjuvant immunotherapy adenocarcinoma group (n=15) and neoadjuvant targeted therapy adenocarcinoma group (n=16)

**Figure 3:**
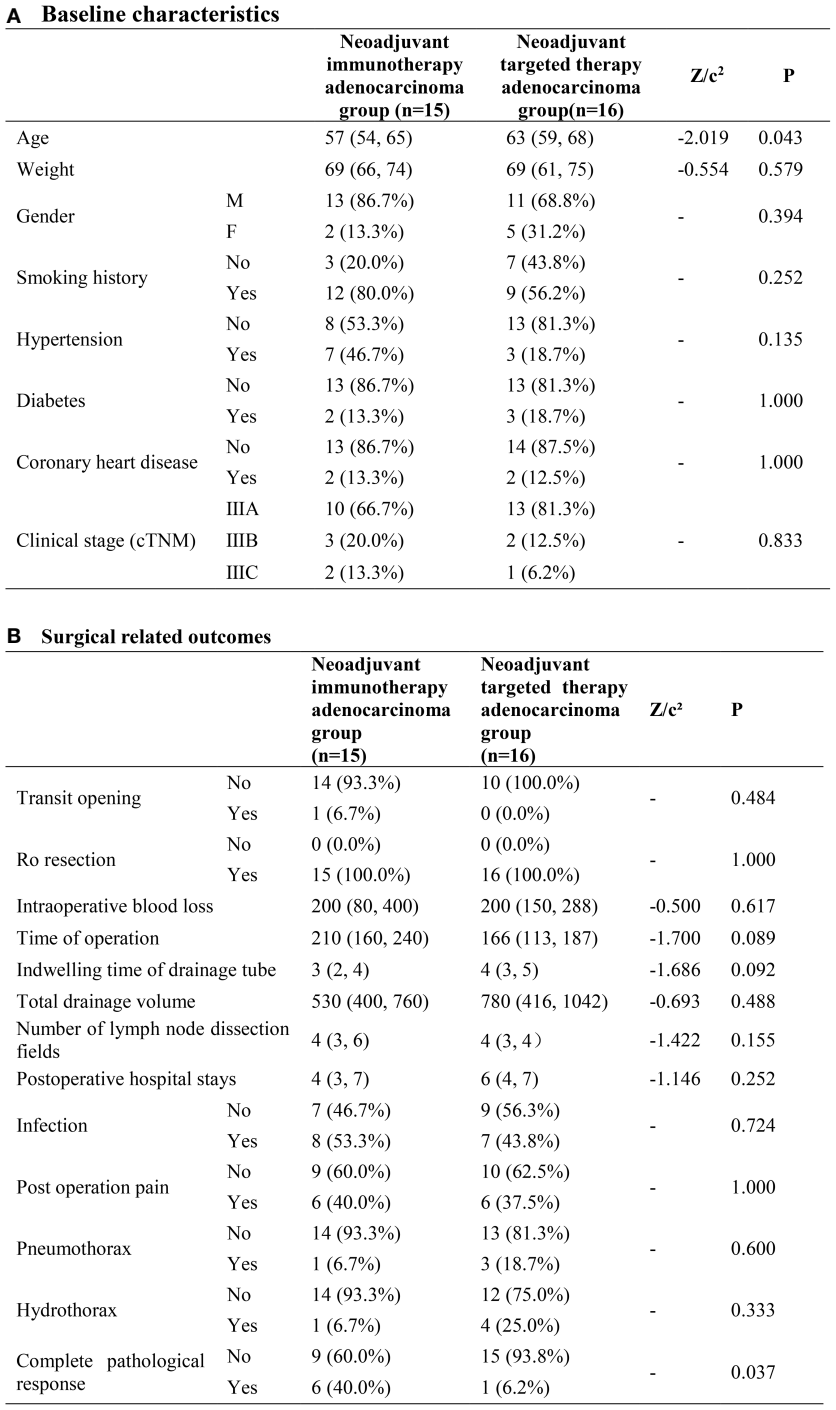
Neoadjuvant immunotherapy adenocarcinoma group and neoadjuvant targeted therapy adenocarcinoma group. **(A)** Baseline characteristics; **(B)** Surgical related outcomes.

As to baseline characteristics, the neoadjuvant immunotherapy adenocarcinoma group had a higher possibility of younger patients compared to the neoadjuvant targeted therapy adenocarcinoma group. (P=0.043) The cPR rate was 40% and 6.2% in the neoadjuvant immunotherapy adenocarcinoma group and neoadjuvant targeted therapy adenocarcinoma group separately, with great heterogeneity (P=0.037). No other significant disparity was observed in the surgical-related outcomes (**Figure 3**).

### Neoadjuvant immunotherapy adenocarcinoma group (n=15) and neoadjuvant chemotherapy adenocarcinoma group (n=10)

**Figure 4:**
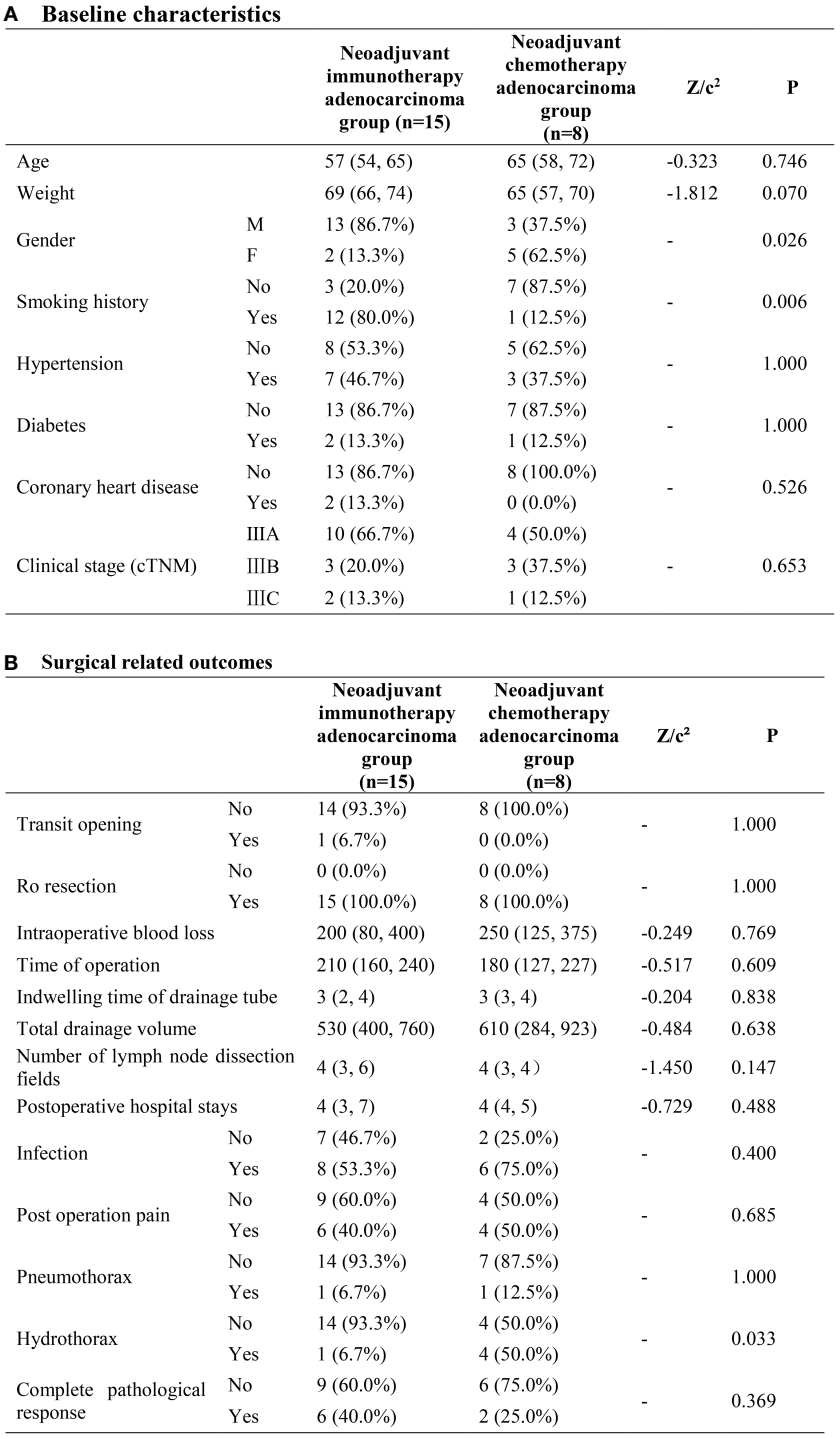
Neoadjuvant immunotherapy adenocarcinoma group and neoadjuvant chemotherapy adenocarcinoma group. **(A)** Baseline characteristics; **(B)** Surgical related outcomes.

In terms of baseline characteristics, no significant disparity was exhibited between the two groups. As to surgical related outcomes, neoadjuvant immunotherapy adenocarcinoma group demonstrated priority with regard to the rate of hydrothorax (6.7%VS 50%, P=0.033). No other difference with statistical significance was observed between the two groups (**Figure 4**).

To conclude, among the six groups, neoadjuvant immunotherapy squamous carcinoma group exhibited advantage over neoadjuvant targeted squamous carcinoma group in terms of the indwelling time of drainage tube and the number of lymph node dissection fields. Neoadjuvant immunotherapy adenocarcinoma group was superior to the neoadjuvant targeted therapy adenocarcinoma group with regard to the cPR. Meanwhile, neoadjuvant immunotherapy adenocarcinoma group overperformed neoadjuvant chemotherapy adenocarcinoma group in the rate of hydrothorax. However, no significant difference was observed between the neoadjuvant immunotherapy squamous carcinoma group and the neoadjuvant chemotherapy carcinoma group.

## Discussion

To the best of our knowledge, there have been limited investigations conducted to elucidate the surgical-related safety and effectiveness of neoadjuvant immunochemotherapy in patients with stage IIIA-IIIC NSCLC who have undergone surgical resection. In our study, we validated the potential advantages of neoadjuvant immunotherapy in terms surgical-related outcomes for advanced stage IIIA-IIIC NSCLC patients.

It is imperative to underscore that, having been categorized into six distinct subgroups, the sample size within each group was relatively modest. Regrettably, this limitation hindered our ability to obtain more profound and satisfying results. For instance, the complete pathological response (cPR) rate was 39.6% in the immunotherapy squamous carcinoma group and 20% in the neoadjuvant chemotherapy squamous carcinoma group, with no detectable heterogeneity. Notably, our neoadjuvant immunotherapy group achieved a cPR rate of 39.6%, significantly higher than the rates of 24.0% in Checkmate-816 (23), 28.6% in NEOSTAR (2021) (22) and 33.3% in Shu et al. (2020) (40). These studies reported respective operation rates of 83.2%, 84.1%, and 96.7%, while our study boasted a flawless 100.0% operation rate. This data strongly reinforces the concept that a higher operation rate correlates positively with a heightened cPR rate following neoadjuvant immunochemotherapy. This suggests that the potential benefits of neoadjuvant immunotherapy may include rendering unresectable NSCLC cases amenable to surgery through tumor shrinkage and preemptive treatment in patients at elevated risk of developing metastatic tumors. Notably, our neoadjuvant targeted squamous carcinoma group achieved a cPR rate of 33%, a figure significantly surpassing the outcomes observed in previous studies (13). This outcome can potentially be attributed in part to preoperative imaging screenings that identified tumor shrinkage.

Of great significance, both cases displaying a complete pathological response were classified as clinical stage IIIA and did not exhibit lymphatic metastasis. Despite the absence of exon 19 deletions or Leu858Arg substitutions in squamous carcinoma, there is a plausible speculation that targeted therapy might hold promise for those patients who are free from lymphatic metastasis (41).

Despite the significant advancements achieved through the use of various drugs as first-line clinical treatments, the diversity in treatment strategies poses significant challenges in comprehending the true extent of the therapeutic benefits of neoadjuvant immunotherapy for NSCLC. Inconsistent outcomes arise from variations in trial endpoints, such as OS, relapse-free survival (RFS), cPR, and major pathological response (mPR), as well as the inclusion of patients with different stages of NSCLC. Moreover, it is essential to acknowledge the significance of Grade 3 or higher irAEs, despite their absence in our study. The mechanism underlying irAEs remains unknown, and the occurrence rates differ considerably across different ICIs (37, 42, 43). The absence of irAEs in our study may illustrate that irAEs generally took place before surgery and impact the suitability of patients for surgical resection. To conclude, conducting further studies on different ICIs is imperative to demonstrate the mechanisms of immunotherapy, understand the relationship between neoadjuvant immunotherapy and irAEs, and establish standardized ICI protocols for different types of NSCLC.

Our study was subject to several inherent limitations. Firstly, being a single-center retrospective study, the sample size was relatively small in our six subgroups which may have introduced sampling errors. Moreover, the diverse range of first-line clinical immunotherapy approaches and the empirical application of neoadjuvant immunotherapy posed significant challenges in standardizing treatment strategies. As a result, the EGFR data and PD-L1 data was not available for all of the patients. Additionally, the follow-up period for prognosis was relatively short for patients who underwent neoadjuvant immunochemotherapy, preventing us from conducting comprehensive survival analyses. In light of these limitations, there is an urgent need for further prospective multicenter clinical studies to provide a more comprehensive understanding of the efficacy and surgical-related safety of neoadjuvant immunotherapy in advanced NSCLC patients.

## Conclusion

In terms of surgical related outcomes, neoadjuvant immunotherapy offered advantages over traditional neoadjuvant chemotherapy and neoadjuvant targeted therapy for patients with the pathological type of adenocarcinoma. With regard to squamous carcinoma, neoadjuvant immunotherapy was superior to neoadjuvant targeted therapy. while more evidence was necessary in order to prove its’ priority over neoadjuvant chemotherapy. Further studies are crucial to address the limitations of our study and contribute to the ongoing debate on this topic.

## Data availability statement

The raw data supporting the conclusions of this article will be made available by the authors, without undue reservation.

## Ethics statement

The studies involving humans were approved by ethics committee of Jinling Hospital. The studies were conducted in accordance with the local legislation and institutional requirements. The participants provided their written informed consent to participate in this study. Written informed consent was obtained from the individual(s) for the publication of any potentially identifiable images or data included in this article.

## Author contributions

QW and YS conceived of the idea and were major contributors in writing the manuscript. M-TX and CQ collected the data. NX, YQ, and CZ performed the statistical analysis. LJ made great comtributions in the statistical analysis in the revision of this manuscript and provided construction ideals. QY performed statistical analysis of this manuscript and made contribution in the revision of this manuscript. All authors contributed to the interpretation of the results and critically reviewed the first draft. All authors contributed to the article and approved the submitted version.
